# The impact of ABO blood type on the prevalence of portal vein thrombosis in patients with advanced chronic liver disease

**DOI:** 10.1111/liv.14404

**Published:** 2020-03-04

**Authors:** Bernhard Scheiner, Patrick G. Northup, Anselm B. Gruber, Georg Semmler, Gerda Leitner, Peter Quehenberger, Johannes Thaler, Cihan Ay, Michael Trauner, Thomas Reiberger, Ton Lisman, Mattias Mandorfer

**Affiliations:** ^1^ Division of Gastroenterology and Hepatology Department of Internal Medicine III Medical University of Vienna Vienna Austria; ^2^ Vienna Hepatic Hemodynamic Laboratory Medical University of Vienna Vienna Austria; ^3^ Center for the Study of Hemostasis in Liver Disease Division of Gastroenterology and Hepatology University of Virginia Charlottesville VA USA; ^4^ Department of Blood Group Serology and Transfusion Medicine Medical University of Vienna Vienna Austria; ^5^ Department of Laboratory Medicine Medical University of Vienna Vienna Austria; ^6^ Division of Hematology and Hemostaseology Department of Medicine I Medical University of Vienna Vienna Austria; ^7^ Surgical Research Laboratory and Section of Hepatobiliary Surgery and Liver Transplantation Department of Surgery University of Groningen University Medical Center Groningen Groningen The Netherlands

**Keywords:** ABO blood type, cirrhosis, portal hypertension, portal vein thrombosis, von Willebrand factor

## Abstract

**Background and aims:**

Non‐O blood type (BT) is a risk factor for thromboses, which has been attributed to its effects on von Willebrand factor (VWF)/factor VIII (FVIII) levels. Although high VWF/FVIII may be risk factors for portal vein thrombosis (PVT) in patients with advanced chronic liver disease (ACLD), the impact of BT on PVT is unknown. We aimed to assess (I) whether non‐O‐BT is a risk factor for PVT and (II) whether non‐O‐BT impacts VWF/factor VIII in patients with ACLD.

**Methods:**

Retrospective analysis comprising two cohorts: (I) “*US*” including all adult liver transplantations in the US in the MELD era and (II) “*Vienna*” comprising patients with a hepatic venous pressure gradient (HVPG) ≥6 mmHg.

**Results:**

(I) The “*US cohort*” included 84 947 patients (non‐O: 55.43%). The prevalence of PVT at the time of listing (4.37% vs 4.56%; *P* = .1762) and at liver transplantation (9.56% vs 9.33%; *P* = .2546) was similar in patients with O‐ and non‐O‐BT. (II) 411 patients were included in the “Vienna cohort” (non‐O: 64%). Mean HVPG was 18(9) mmHg and 90% had an HVPG ≥10 mmHg. Patients with non‐O‐BT had slightly increased VWF levels (318(164)% vs 309(176)%; *P* = .048; increase of 23.8%‐23.9% in adjusted analyses), but this difference was driven by patients with less advanced disease. However, non‐O‐BT explained only 1% of the variation in VWF and had no effect on FVIII.

**Conclusions:**

Although non‐O‐BT impacts VWF in patients with early stage ACLD, its contribution to VWF variation is considerably smaller than in the general population. Moreover, non‐O‐BT had no impact on FVIII. These findings may explain the absence of an association between non‐O‐BT and PVT in patients with advanced cirrhosis.

**Key points**

- Non‐O blood type is a risk factor for thromboses in the general population.
- This association has been attributed to increased von Willebrand Factor (VWF)/factor VIII levels in subjects with non‐O blood type.
- The impact of blood type on portal vein thrombosis (PVT) in patients with advanced chronic liver disease (ACLD) is unknown.
- In this study, the prevalence of PVT was comparable between patients with O and non‐O blood type undergoing liver transplantation.
- The contribution of non‐O blood type to VWF variation was considerably smaller than in the general population and was limited to patients with early stage ACLD.
- Non‐O blood type had no effect on factor VIII levels in ACLD patients.
- These findings may explain the absence of an association between blood type and PVT in advanced cirrhosis.

## 1 INTRODUCTION

As a consequence of abnormal routine coagulation tests and thrombocytopenia, cirrhosis has long been considered an acquired bleeding disorder.^1^ In fact, the liver plays a central role in coagulation and plasma levels of most procoagulant factors are significantly reduced in patients with cirrhosis. However, these changes are balanced by decreased levels of anticoagulant proteins^2^ and highly elevated levels of the platelet adhesive protein von Willebrand factor (VWF).^3^ Therefore, patients with cirrhosis are nowadays considered to have a rebalanced haemostatic equilibrium.^4^ However, when compared to liver‐healthy subjects, this equilibrium seems to be instable and easily tips in one direction, which may lead to bleeding or thrombosis.^5^, ^6^ However, while clinically relevant non‐portal hypertensive bleeding is rare, the prevalence/incidence of portal vein thrombosis (PVT) in patients with advanced cirrhosis is considerably high.^7^ PVT is a clinically relevant complication in patients with cirrhosis,^7^ especially in the liver transplant waiting list setting. While there is an ongoing debate regarding a potential causal relationship between PVT and deterioration of liver function,^8^ extensive PVT has been shown to worsen transplant outcomes in an analysis based on the OPTN/UNOS data^9^ or may even preclude liver transplantation. Moreover, the risk of venous thromboembolism (VTE; ie, deep vein thrombosis and pulmonary embolism) is significantly increased in patients with cirrhosis.^10^ As a result, cirrhosis may even be considered as a prothrombotic condition, with thrombophilic changes such as elevated VWF/factor VIII levels potentially contributing to the high risk of thrombotic events.^4^

The multimeric glycoprotein VWF is released from endothelial cells upon activation and is cleaved by the ADAMTS13 protease into smaller, haemostatically less potent multimers.^11^ Apart from portal hypertension,^12^ several other factors influencing VWF levels have been identified. For instance, in the general population/studies not focusing on chronic liver disease (CLD), VWF increases with age^13^ or in the presence of the metabolic syndrome (MetS).^14^ Moreover, VWF levels are determined by ABO blood type. VWF and factor VIII levels are about 25% higher in non‐O individuals, as compared to O individuals,^15^ which translates into a clinically significantly increased risk of VTE (eg, incidence rate ratio of 1.8 and population attributable risk of 32%),^15^, ^16^ and to a smaller extent, arterial thrombosis (in particular, myocardial infarction: incidence rate ratio of 1.1 and population attributable risk of 6%).^16^, ^17^ Moreover, some evidence links VWF/factor VIII levels with incident PVT in patients with cirrhosis.^18^

However, in patients with clinically significant portal hypertension (CSPH), VWF levels are highly elevated, most likely as a result of portal hypertension^19^ and bacterial translocation‐induced inflammation.^12^ Accordingly, it is unclear if, and to what extent, ABO blood type impacts on VWF/factor VIII levels in patients with cirrhosis. Furthermore, the effect of ABO blood type on the development of PVT, the most common thrombotic event in patients with cirrhosis, has yet to be studied. Prevention of PVT development with prophylactic enoxaparin seems to be effective and safe in patients awaiting liver transplantation.^20^ Identification of patients at particularly high risk for PVT may facilitate patient selection for future studies on prophylactic anticoagulation.

Therefore, we aimed to analyse the impact of ABO blood type on (I) the development of PVT and (II) VWF/factor VIII levels in patients with advanced chronic liver disease (ACLD).

## 2 PATIENTS AND METHODS

### 2.1 Study design and population

This retrospective analysis included two cohorts:

(I) The “*US cohort*” included all adult (12 years old and above) liver transplantations in the US in the model for end‐stage liver disease (MELD) era up to the end of 2017 (02/2002‐12/2017). Data were obtained through the Organ Procurement and Transplantation Network (OPTN) and the data set supplied by the United Network for Organ Sharing (UNOS). Liver re‐transplantations, transplantations for acute liver failure and entries with missing information on blood type were excluded. Moreover, patients with hepatocellular carcinoma (HCC) and/or transjugular portosystemic shunt (TIPS) were excluded from the data set for the main analyses. Finally, the analyses were repeated after re‐including these patients.

Blood type A subgroups (A1, A1B, A2 and A2B) were referred to as blood type A in all analyses.

(II) The “*Vienna cohort*” comprised prospectively characterised patients with ACLD who underwent hepatic venous pressure gradient (HVPG) measurement at the Medical University of Vienna between 01/2006 and 02/2018 and had a HVPG ≥6 mmHg and available information on ABO blood type and plasma VWF levels. Patients with active bacterial infection or hepatocellular carcinoma (HCC) were excluded. The final cohort comprised 411 patients.

### 2.2 Evaluation of portal vein thrombosis

In the “*US cohort*,” portal vein thrombosis was evaluated at the time of listing as well as at the time of liver transplantation. The required information was independently reported to the OPTN by each transplant centre as part of the routine transplantation waiting list registry and the transplant event registry.

### 2.3 HVPG measurement

Hepatic venous pressure gradient was measured in clinical routine for diagnostic or prognostic purposes or HVPG‐guided non‐selective betablocker therapy,^21^ as supported by the Austrian consensus recommendations for the treatment of portal hypertension.^22^, ^23^, ^24^ HVPG measurements were performed in the absence of portal pressure‐lowering medications (ie, non‐selective beta blockers and nitrates) or transjugular intrahepatic portosystemic shunt (TIPS) and according to a standardised protocol, as previously described.^25^, ^26^ HVPG values ≥10 mmHg denoted CSPH.

### 2.4 Assessment of VWF and factor VIII levels

Labouratory tests in the “*Vienna cohort*” were performed using blood samples obtained at the time of HVPG measurement. VWF antigen levels were measured by a latex agglutination assay (STA LIATEST vWF, Diagnostica Stago), while factor VIII activity was assessed by a one‐stage clotting assay using a fully automated CS‐5100 coagulation analyser (Sysmex). Plasma protein C and antithrombin activity were measured using chromogenic assays.

### 2.5 Statistical analyses

Statistical analyses were performed using IBM SPSS Statistics 25 (IBM), GraphPad Prism 8 (GraphPad Software) and SAS 9.4 (SAS Institute). Categorical variables were reported as numbers (n) and proportions (%) of patients and continuous variables as mean ± standard deviation or median (interquartile range [IQR]), as appropriate. Differences in categorical variables were analysed for using the Chi‐squared test. Student's *t* tests/analyses of variance (ANOVA) with generalised linear modelling using the least square means technique or Mann‐Whitney *U* tests/Kruskal Wallis one‐way ANOVA were used to compare continuous variables. Correlations were analysed by calculating Spearman's correlation coefficients, which were further compared by Fisher transformation. Simple and multiple linear regression analyses were performed to evaluate factors independently associated with VWF/factor VIII levels. Variables showing a trend in univariate analysis (*P* < .1) as well as the factor of interest (non‐O blood type) were included into the multivariate models. Multicollinearity was detected by variable inflation factor (VIF). Accordingly, either decompensated cirrhosis and MELD, or Child‐Turcotte‐Pugh (CTP) score were included as covariates. A *P* ≤ .05 was considered statistically significant.

### 2.6 Ethics

The sub‐study based on the “*Vienna cohort*” was conducted in accordance with the Declaration of Helsinki and approved by the institutional review board (IRB) of the Medical University of Vienna (No. 1446/2018), which waived the requirement of a written informed consent for this retrospective analysis. The University of Virginia does not require IRB approval for OPTN data set analyses and no study data related to the “*Vienna cohort*” were revealed to the US investigator.

## 3 RESULTS

### 3.1 Study populations

**FIGURE 1:**
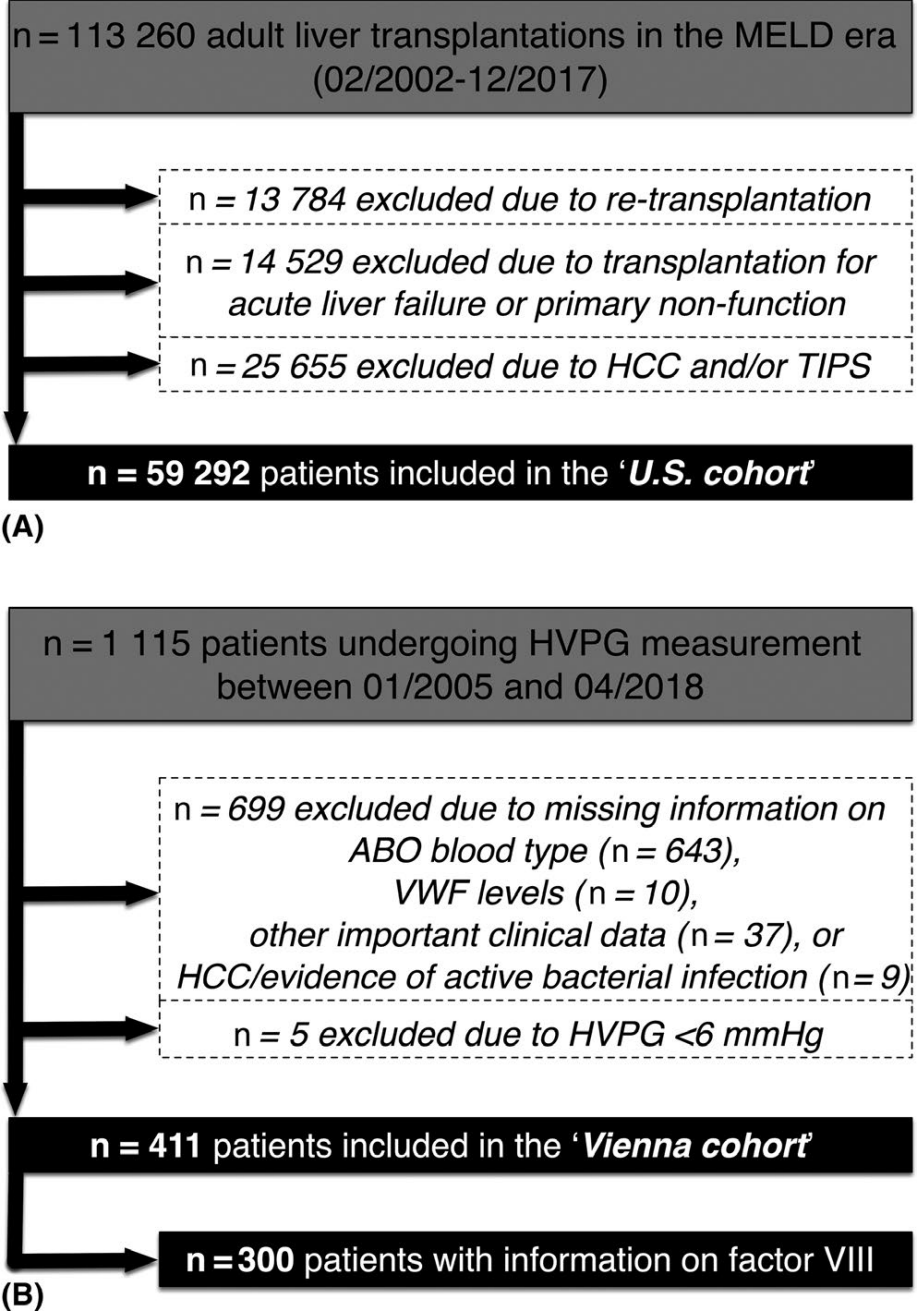
Flow chart of the A “*US cohort*” and the B “*Vienna cohort*.” HVPG hepatic venous pressure gradient; MELD model for end‐stage liver disease

(I) In total, and after excluding patients undergoing re‐transplantation (n = 13 784) and transplantations for acute liver failure or primary graft non‐function (n = 14 529), as well as patients with HCC and/or TIPS, 59 292 patients were included in the “*US cohort*” (Figure 1). The analyses were repeated after re‐including 25 655 patients with HCC and/or TIPS resulting in a study population of 84 947 patients.

(II) Within the study period, 1115 individual patients underwent HVPG measurement at the Vienna Hepatic Hemodynamic Lab (Figure 1). According to the in‐ and exclusion criteria, 690 patients were excluded because of missing information on ABO blood type (n = 643), VWF (n = 10), important clinical data (n = 37), or HCC/evidence of active bacterial infection (n = 9). Additionally, five patients were excluded owing to HVPG < 6 mmHg. Finally, 411 well‐characterised patients were included in the “*Vienna cohort.”*.

### 3.2 Patient characteristics of the “US cohort” according to ABO blood type

Overall, the majority of patients was male (63.2%) with a mean age of 52.55 ± 11.68 years. While 26 481 patients had blood type O, 21 902 had A, 7915 B and 2994 patients harboured blood type AB. Detailed patient characteristics of the “*US cohort*” stratified by blood type are displayed in Table S1.

As this study aimed to evaluate differences between O vs non‐O individuals, a comparison of baseline characteristics between these two groups is shown in Table 1. While 55.34% of patients (n = 32 811) had non‐O blood type, 44.66% (n = 26 481) harboured O blood type. As a result of the large sample size, aetiology of liver disease was statistically significantly different between the two groups (*P* < .0001), however, the differences were small and judged not to be clinically significant (maximum between group difference: 1.06%). MELD score at the time of liver transplantation was statistically significantly higher in patients with blood type O (24.24 ± 10.45), when compared to non‐O individuals (23.22 ± 10.18 points; *P* < .0001). This may partially be explained by differences in waiting times for liver transplantation (202 ± 374 vs 188 ± 355 days; *P* = .0459), since MELD at listing showed an even smaller difference between blood types (24.24 ± 10.45 vs 23.33 ± 10.18; *P* < .0001). Importantly, despite being statistically significant, the differences in MELD were only modest. Moreover, patients with blood type O showed a lower prevalence of diabetes mellitus (22.40% vs 23.26%; *P* = .0132), but again, this difference was not considered clinically significant. Importantly, the proportions of overweight and obese patients as well as other baseline characteristics were well balanced between the two groups.

**TABLE 1:** Comparison of patient characteristics between patients with O and non‐O blood types in the “*US cohort*” (excluding patients with HCC and/or TIPS)

Patient characteristics	All patients, n = 59 292	O, n = 26 481	Non‐O, n = 32 811	*P* value
Age, y	52.55 ± 11.68	52.43 ± 11.76	52.64 ± 11.62	.1274
Sex				
Male	37 546 (63.32%)	16 707 (63.09%)	20 839 (63.51%)	.2895
Female	21 746 (36.68)	9774 (36.91%)	11 972 (36.49%)	
Overweight^a^	40 347 (68.05%)	18 086 (68.30%)	22 261 (67.85%)	.2407
Obese^b^	20 299 (34.24%)	9069 (34.25%)	11 230 (34.23%)	.9575
Diabetes mellitus	13 564 (22.88%)	5 932 (22.4%)	7 632 (23.26%)	.0132
Aetiology				
Viral	21 507 (36.27%)	9613 (36.30%)	11 894 (36.25%)	<.0001
ALD	10 288 (17.35%)	4522 (17.08%)	5766 (17.57%)	
NAFLD or cryptogenic	9 834 (16.59%)	4302 (16.25%)	5532 (16.86%)	
Other	17 663 (29.79%)	8044 (30.38%)	9619 (29.32%)	
Time on the waiting list	194 ± 364	202 ± 374	188 ± 355	.0459
MELD at listing, points	20.72 ± 9.64	21.06 ± 9.79	20.45 ± 9.52	<.0001
MELD at transplant, points	23.74 ± 10.31	24.24 ± 10.45	23.33 ± 10.18	<.0001
PVT at listing	2521 (4.25%)	1087 (4.10%)	1434 (4.37%)	.1110
PVT at transplant	5024 (8.47%)	2223 (8.39%)	2801 (8.54%)	.5369

Abbreviations: ALD, alcoholic liver disease; BMI, body mass index; HCC, hepatocellular carcinoma; MELD, model for end‐stage liver disease; NAFLD, non‐alcoholic fatty liver disease; PVT, portal vein thrombosis; TIPS, transjugular intrahepatic portosystemic shunt.

^a^ BMI > 25 kg × m^−2^.

^b^ BMI > 30 kg × m^−2^.

### 3.3 Prevalence of PVT at the time of listing for liver transplantation and transplantation in the “US cohort”

**FIGURE 2:**
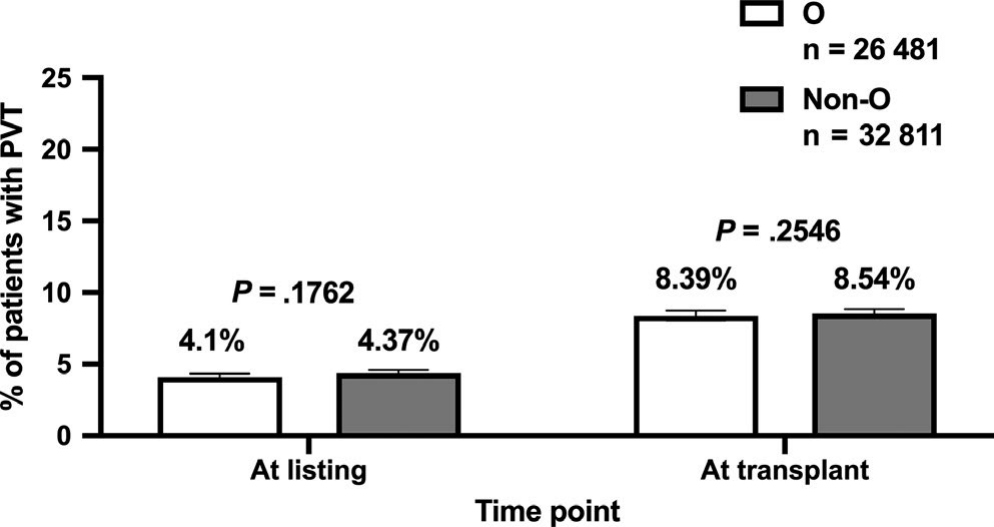
Comparison of portal vein thrombosis (PVT) prevalence at the time of listing for liver transplantation as well as at the time of transplantation between patients with O vs non‐O blood types in the “*US cohort*” (excluding patients with hepatocellular carcinoma [HCC] and/or transjugular intrahepatic portosystemic shunt [TIPS])

At the time of listing for liver transplantation, 4.25% of patients without HCC and/or TIPS had a PVT and the rate of PVT was similar in individuals with O vs non‐O blood type (4.1% [1087/26 481]) vs 4.37% [1434/32 811]; *P* = .111; Table 1; Figure 2). The PVT prevalence increased to 8.47% at the time of liver transplantation, however, again, the PVT rate was similar between blood type O vs non‐O (8.39% [2223/26 481] vs 8.54% [2801/32 811]; *P* = .5369). To assess whether the relationship between blood type and PVT prevalence is modified by the severity of liver disease, we excluded patients with HCC and/or TIPS and stratified the “*US cohort*” according to MELD score <15, 15‐20 and >20 points at listing. The prevalence of PVT at listing was comparable between patients with O vs non‐O blood type throughout all MELD strata: <15: 3.73% vs 3.92% (*P* = .517), 15‐20: 4.4% vs 4.69% (*P* = .384) and >20 points: 4.17% vs 4.49% (*P* = .208).

Finally, we re‐included the patients with HCC and/or TIPS, which did not affect our results regarding the impact of ABO blood type on PVT (Tables S2 and S3).

### 3.4 Patient characteristics of the “Vienna cohort” according to ABO blood type

The majority of patients was male (70%) with a mean age of 54.1 ± 11.4 years (Table 2). Viral hepatitis (38%) and alcoholic liver disease (ALD; 35%) were the most common aetiologies. While 16% (64/411) of patients were Child‐Turcotte‐Pugh (CTP) score stage A, 63% (259/411) were classified as CTP B and 21% (88/411) as CTP C. Median MELD was 11^9^ points. According to inclusion criteria, all patients had portal hypertension and 90% (370/411) of patients were diagnosed with CSPH. While more than two thirds (73% [301/411]) of patients had varices, 47% (194/411) had ascites, 28% (115/411) had a history of or current overt hepatic encephalopathy and every fifth patient (19% [80/411]) had a history of variceal bleeding. In the “Vienna cohort,” almost two thirds of patients (64% [264/411]) presented with a non‐O blood type, while 36% (147/411) of patients had O blood type.

**TABLE 2:** Characteristics of the “*Vienna cohort*” at the time of HVPG measurement and comparison between patients with O and non‐O blood types

Patient characteristics	All patients, n = 411	O, n = 147	Non‐O, n = 264	*P* value
Age, y	54.1 ± 11.4	54.1 ± 11.2	54.1 ± 11.5	.954
Sex				
Male	286 (70%)	100 (68%)	186 (70%)	.608
Female	125 (30%)	47 (32%)	78 (30%)	
BMI, kg × m^−2^	26 (6.4)	25.7 (5.7)	26.2 (7.2)	.3
Overweight^a^	237 (58%)	82 (56%)	155 (59%)	.564
Obesity^b^	88 (21%)	23 (16%)	65 (25%)	.034
Arterial hypertension	112 (27%)	36 (25%)	76 (29%)	.348
Diabetes mellitus	92 (22%)	31 (21%)	61 (23%)	.638
Hypertriglyceridemia^c^	31 (8%)	10 (7%)	21 (8%)	.657
Hypercholesterolaemia^d^	45 (11%)	17 (12%)	28 (11%)	.796
Aetiology				
Viral	156 (38%)	60 (41%)	96 (36%)	.679
ALD	144 (35%)	50 (34%)	94 (36%)	
NAFLD or cryptogenic	69 (17%)	21 (14%)	48 (18%)	
Other	42 (10%)	16 (11%)	26 (10%)	
Varices	301 (73%)	107 (73%)	194 (73%)	.879
Decompensated	261 (64%)	96 (65%)	165 (63%)	.571
History of variceal bleeding	80 (19%)	33 (22%)	47 (18%)	.254
Ascites				
None	217 (53%)	75 (51%)	142 (54%)	.849
Mild	144 (35%)	54 (37%)	90 (34%)	
Severe	50 (12%)	18 (12%)	32 (12%)	
History of/current overt hepatic encephalopathy	115 (28%)	44 (30%)	71 (27%)	.511
HVPG, mmHg	18 (9)	18.2 (8)	18 (9)	.084
HVPG 6‐9 mmHg	41 (10%)	13 (9%)	28 (11%)	.76
HVPG 10‐15 mmHg	102 (25%)	35 (24%)	67 (16%)	
HVPG ≥ 16 mmHg	268 (65%)	99 (67%)	169 (64%)	
MELD, points	11 (5)	12 (6)	11 (6)	.084
CTP score, points	8 (2)	8 (2)	8 (2)	.189
A	64 (16%)	20 (14%)	44 (17%)	.713
B	259 (63%)	95 (65%)	164 (62%)	
C	88 (21%)	32 (22%)	56 (21%)	
Platelet count, G × L^−1^	98 (72)	94 (77)	99 (70)	.881
Albumin, g × L^−1^	35.5 (8.2)	35.5 (8)	35.5 (8.3)	.959
Bilirubin, mg × dL^−1^	1.3 (1.47)	1.25 (1.85)	1.31 (1.2)	.857
INR	1.3 (0.3)	1.3 (0.2)	1.3 (0.3)	.876
AST, U × L^−1^	52 (46)	54 (56)	50 (38)	.502
ALT, U × L^−1^	35 (42)	36 (42)	35 (41)	.725
GGT, U × L^−1^	104 (118)	104 (120)	104 (119)	.691
CRP, mg × L^−1^	0.28 (0.61)	0.34 (0.59)	0.27 (0.62)	.208
VWF, %	313 (167)	309 (176)	318 (164)	.048
Factor VIII^e^, %	199 (6)	198 (85)	199 (88)	.882
Protein C^f^, %	53 (33)	53 (29)	54 (36)	.845
Antithrombin^g^, %	64 (28)	61 (23)	65 (31)	.528
Factor VIII/protein C ratio^h^	3.74 (2.74)	3.74 (2.39)	3.75 (2.82)	.696

Abbreviations: ALD, alcoholic liver disease; ALT, alanine transaminase; AST, aspartate transaminase; BMI, body mass index; CRP, C‐reactive protein; CTP, Child‐Turcotte‐Pugh score; GGT, gamma‐glutamyltransferase; HVPG, hepatic venous pressure gradient; INR, international normalised ratio; MELD, model for end‐stage liver disease; NAFLD, non‐alcoholic fatty liver disease; VWF, von Willebrand factor antigen.

^a^ BMI > 25 kg × m^−2^.

^b^ BMI > 30 kg × m^−2^.

^c^ Triglycerides > 150 mg × dL^−1^.

^d^ Total cholesterol > 200 mg × dL^−1^.

^e^ Available in n = 300 patients.

^f^ Available in n = 358 patients.

^g^ Available in n = 361 patients.

^h^ Available in n = 249 patients.

**FIGURE 3:**
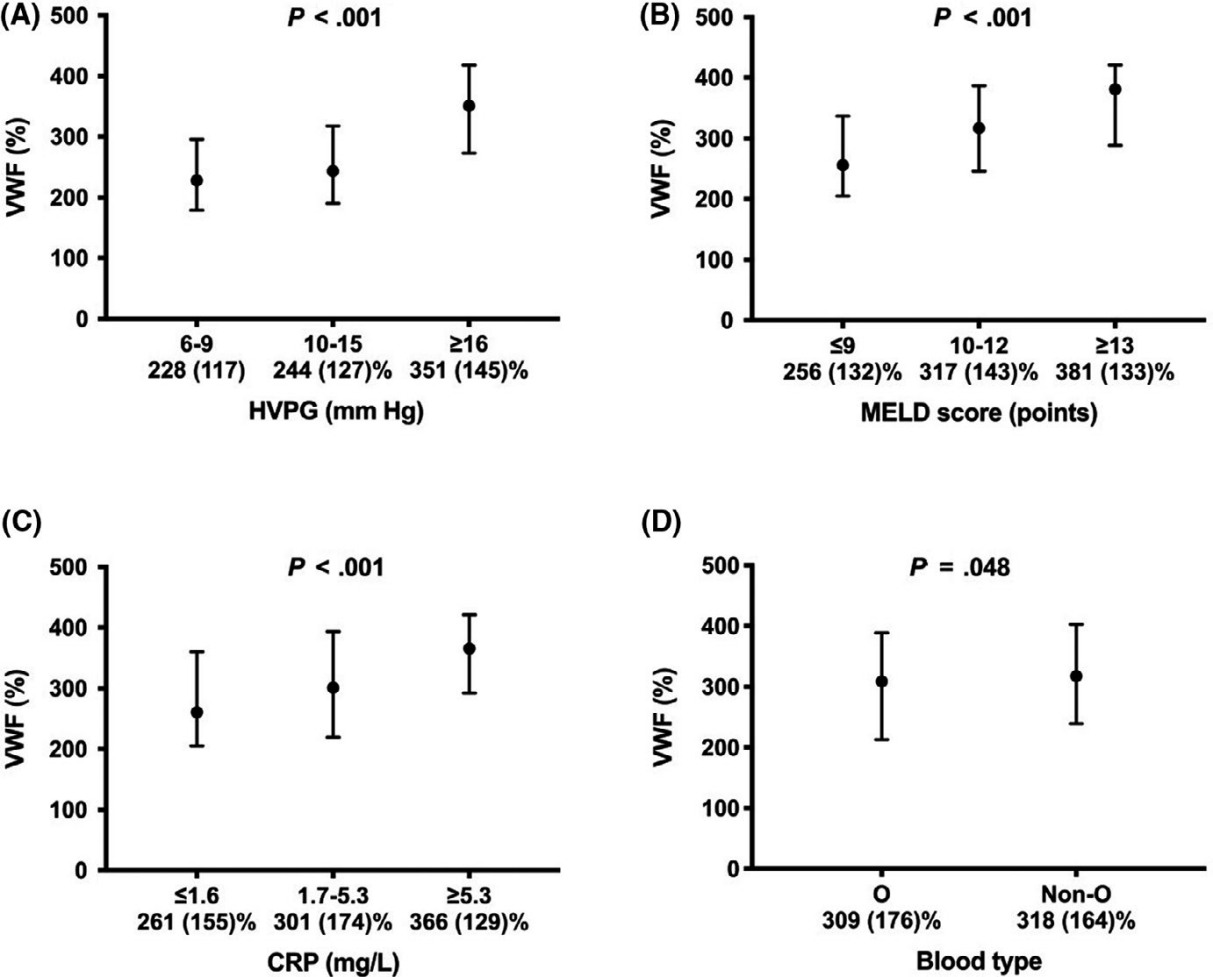
VWF levels throughout different (A) HVPG strata, (B) MELD score and (C) CRP terciles, as well as (D) blood types of the “*Vienna cohort*.” VWF von Willebrand factor antigen; HVPG hepatic venous pressure gradient; MELD model for end‐stage liver disease; CRP C‐reactive protein

Median overall VWF level was 313 (IQR: 167)% with slightly, but statistically significantly higher levels in non‐O blood type individuals (318 [IQR: 164]%), when compared to O patients (309 [IQR: 176]; *P* = .048; Figure 3). In contrast, factor VIII levels were similar in O (198 [IQR: 85]) and non‐O (199 [IQR: 88]; *P* = .882) patients.

Moreover, plasma activities of protein C (information available in n = 358) and antithrombin (n = 361), as well as factor VIII/protein C ratio (n = 249) did not differ between patients with O and non‐O blood type. Except for trends towards a slightly higher HVPG and MELD score in blood type O patients (*P* = .084 for both), all other baseline characteristics were well balanced between the groups.

VWF levels were significantly higher in blood type non‐O patients with low MELD (<10 points) or subclinical portal hypertension (HVPG 6‐9 mmHg), while no differences were observed in patients with HVPG values of 10‐15 mmHg and profound portal hypertension (HVPG ≥ 16 mmHg), or patients with MELD scores of 10‐15 and >15 points (Tables S4 and S5). When stratifying by CTP score stage, there was a numerical difference in CTP A patients, however, this trend did not attain statistical significance. A similar pattern was observed when analyzing factor VIII levels, with trends towards higher factor VIII levels in blood type non‐O patients with CTP A, MELD <10 points, or subclinical portal hypertension (HVPG 6‐9 mmHg). However, these trends did not attain statistical significance. Of note, factor VIII values were very similar between O and non‐O patients with more liver disease/portal hypertension.

### 3.5 Adjusted and unadjusted analyses on factors associated with VWF levels in the “Vienna cohort”

In unadjusted analysis, VWF levels showed a positive association with age, ALD as underlying aetiology, HVPG and factors closely related to portal hypertension (ie, varices and hepatic decompensation) and indicators of hepatic dysfunction (MELD and CTP score) (Table 3). In addition, VWF was linked with liver enzyme levels (aspartate transaminase [AST] and gamma‐glutamyltransferase [GGT]), systemic inflammation (C‐reactive protein [CRP]) and non‐O blood type (unstandardised regression coefficient [B]: 18.7; *P* = .047).

**TABLE 3:** Simple (A) and multiple linear regression analysis (B model including decompensated cirrhosis and MELD; C model including CTP score) of factors associated with VWF levels in the “*Vienna cohort*”

Patient characteristics	A		B		C	
	B	*P* value	B	*P* value	B	*P* value
Age, y	1.09	.006	1.2	<.001	1.13	.001
Male sex	1.37	.889	—	—	—	—
BMI, kg × m^−2^	0.107	.907	—	—	—	—
Overweight^a^	−5.98	.512	—	—	—	—
Obesity^b^	12.26	.264	—	—	—	—
Arterial hypertension	−5.40	.594	—	—	—	—
Diabetes mellitus	−4.43	.682	—	—	—	—
Hypertriglyceridemia^c^	−9.35	.583	—	—	—	—
Hypercholesterolaemia^d^	17.68	.22	—	—	—	—
ALD, vs other aetiologies	50.9	<.001	14.9	.109	16.1	.066
Varices	40.5	<.001	8.1	.386	9.39	.306
Decompensated cirrhosis	67.9	<.001	22.5	.023	—	—
HVPG, mmHg	5.95	<.001	3.75	<.001	3.54	<.001
MELD, points	6.81	<.001	3.11	.004	—	—
CTP score, points	21.8	<.001	—	—	13.5	<.001
Platelet count, G × L^−1^	−0.024	.736	—	—	—	—
AST, U × L^−1^	0.121	.012	0.12	.005	0.12	.005
ALT, U × L^−1^	0.016	.572	—	—	—	—
GGT, U × L^−1^	0.072	.019	0.054	.045	0.06	.023
CRP, mg × L^−1^	4.58	<.001	1.79	.013	1.32	.069
Non‐O	18.7	.047	23.9	.003	23.8	.003

Abbreviations: ALD, alcoholic liver disease; ALT, alanine transaminase; AST, aspartate transaminase; BMI, body mass index; CRP, C‐reactive protein; CTP, Child‐Turcotte‐Pugh score; GGT, gamma‐glutamyltransferase; HVPG, hepatic venous pressure gradient; MELD, model for end‐stage liver disease; VWF, von Willebrand factor.

^a^ BMI > 25 kg × m^−2^.

^b^ BMI > 30 kg × m^−2^.

^c^ Triglycerides > 150 mg × dL^−1^.

^d^ Total cholesterol > 200 mg × dL^−1^.

After adjusting for potentially confounding factors in multivariate models including either hepatic decompensation and MELD, or CTP score, the absolute differences in VWF levels between patients with non‐O and O blood type ranged from 23.8% to 23.9% (*P* = .003 for both models). Furthermore, age (years; B: 1.2; *P* = .001/B: 1.13; *P* = .001), decompensated cirrhosis (B: 22.5; *P* = .023), HVPG (mmHg; B: 3.75; *P* < .001/B: 3.54; *P* < .001), MELD (points; B: 3.11; *P* = .004) and CTP score (points; B: 13.5; *P* < .001), AST; U × L^−1^; B: 0.12 and *P* = .005 for both models), GGT (U × L^−1^; B: 0.054; *P* = .045/B: 0.06; *P* = .023) and C‐reactive protein (CRP; mg × L^−1^; B: 1.79; *P* = .013/B: 1.32; *P* = .069) were independently associated with VWF levels.

VWF levels across HVPG strata as well as MELD and CRP terciles are depicted in Figure 3.

### 3.6 Adjusted and unadjusted analyses on factors associated with factor VIII levels in the “Vienna cohort”

Factor VIII levels showed positive associations with age, components of the MetS (arterial hypertension, hypertriglyceridemia and hypercholesterolaemia), indicators of hepatic dysfunction (MELD and CTP score), platelet count, indicators of liver injury (AST, ALT and GGT) and systemic inflammation (CRP) (Table 4). Interestingly, factor VIII showed no correlation with portal hypertension or its clinical sequalae (ie, varices and hepatic decompensation), or non‐O blood type.

**TABLE 4:** Simple (A) and multiple linear regression analysis (B model including decompensated cirrhosis and MELD; C model including CTP score) of factors associated with factor VIII levels in a subgroup of 300 patients of the “*Vienna cohort*”

Patient characteristics	A		B		C	
	B	*P* value	B	*P* value	B	*P* value
Age, y	0.883	.017	0.832	.019	0.781	.027
Male sex	2.71	.766	—	—	—	—
BMI, kg × m^−2^	0.049	.954	—	—	—	—
Overweight^a^	−6.84	.407	—	—	—	—
Obesity^b^	6.32	.529	—	—	—	—
Arterial hypertension	29.6	.001	18.9	.026	20.8	.013
Diabetes mellitus	11.7	.229	—	—	—	—
Hypertriglyceridemia^c^	42.4	.003	19.9	.146	21.5	.114
Hypercholesterolaemia^d^	35.7	.007	16.1	.205	14	.259
ALD, vs other aetiologies	3.86	.644	—	—	—	—
Varices	−8.63	.337	—	—	—	—
Decompensated cirrhosis	14.2	.102	6.14	.488	—	—
HVPG, mmHg	−0.142	.834	—	—	—	—
MELD, points	2.74	.009	1.92	.076	—	—
CTP score, points	6.21	.003	—	—	5.06	.014
Platelet count, G × L^−1^	0.412	<.001	0.285	<.001	0.275	<.001
AST, U × L^−1^	0.159	<.001	0.107	.003	0.111	.002
ALT, U × L^−1^	0.087	<.001	—	—	—	—
GGT, U × L^−1^	0.095	<.001	0.041	.095	0.042	.086
CRP, mg × L^−1^	2.14	.001	9.52	.152	8.44	.207
Non‐O	1.62	.848	3.11	.411	2.7	.719

Abbreviations: ALD, alcoholic liver disease; ALT, alanine transaminase; AST, aspartate transaminase; BMI, body mass index; CRP, C‐reactive protein; CTP, Child‐Turcotte‐Pugh score; GGT, gamma‐glutamyltransferase; HVPG, hepatic venous pressure gradient; MELD, model for end‐stage liver disease.

^a^ BMI > 25 kg × m^−2^.

^b^ BMI > 30 kg × m^−2^.

^c^ Triglycerides > 150 mg × dL^−1^.

^d^ Total cholesterol > 200 mg × dL^−1^.

The following factors were independently linked to factor VIII levels: age (years; B: 0.832; *P* = .019/B: 0.781; *P* = .027), arterial hypertension (B: 18.9; *P* = .026/B: 20.8; *P* = .013), CTP score (points; B: 5.06; *P* = .014), PLT (B: 0.285; *P* < .001/B: 0.275; *P* < .001) and AST (B: 0.107; *P* = .003/B: 0.111; *P* = .002).

### 3.7 Correlation between VWF and factor VIII levels in the “Vienna cohort”

Von Willebrand factor and factor VIII showed a correlation of moderate strength (ρ = 0.466; *P* < .001) (Table S6). Moreover, the correlations attained statistical significance throughout all CTP, MELD and HVPG groups/strata. We did not observe statistically significant differences in correlations when comparing the different groups/strata using Fisher transformation, even before adjusting for multiple comparisons.

### 3.8 Comparison of patient characteristics between the “US cohort” and the “Vienna cohort”

After excluding patients with HCC and/or TIPS, the male predominance was less pronounced in the “*US cohort*,” as compared to the “*Vienna cohort*,” which may be related to the lower prevalence of ALD in the “*US cohort*” (Table S7). In contrast, patients in the “*US cohort*” were more commonly overweight or obese. The most striking difference was the higher severity of underlying hepatic dysfunction in the “*US cohort*,” as evidenced by a MELD score at the time of listing (20.7 ± 9.6 points), which was considerably higher than the MELD at the time of HVPG measurement in the “*Vienna cohort*” (11.7 ± 4.0 points; *P* < .0001).

## 4 DISCUSSION

Current European guidelines recommend extensive testing for underlying prothrombotic risk factors in patients with PVT without underlying liver disease to guide decisions on long‐term anticoagulation. In contrast, in the setting of ACLD, the relevance of thrombophilia which is not related to the severity of liver disease itself is less clear.^7^ Previous studies indicate that inherited (ie, factor V Leiden and prothrombin G20210A mutation) and acquired factors may be of relevance,^7^ however, none of the inherited factors was associated with incidental PVT in the largest longitudinal study to date.^27^

In the general population, the impact of ABO blood type on VTE is well established. In a study assessing 1.5 million blood donors, for instance, the risk of VTE was increased by 80% in patients with non‐O blood type.^16^ In contrast, the association between ABO blood type and PVT has yet to be investigated. In our large cohort of patients undergoing liver transplantation (“*US cohort*”), the prevalence of PVT at the time of listing as well as at the time of transplantation was similar between O and non‐O individuals. Importantly, owing to the enormous sample size of the “*US cohort*,” we can basically rule out type II error, as we were able to assess even very small differences. For instance, a 10% decrease in the prevalence of portal vein thrombosis in patients with O blood type at the time of listing (ie, from 4.48% to 4.032%) would have been detected with a statistical power of more than 99%. Finally, the OPTN/UNOS data have been used in a series of studies reporting information on PVT,^28^, ^29^, ^30^, ^31^, ^32^, ^33^, ^34^ and thus, seems to be suitable for the purpose of our study.

The well‐documented prothrombotic state observed in liver‐healthy non‐O subjects is attributed to the impact of ABO blood type on VWF, and consequently, factor VIII levels.^35^ Even though the underlying mechanisms have yet to be fully elucidated,^36^ the effects of ABO blood type on VWF and factor VIII levels seem to arise from differences in VWF clearance.^37^ In‐vitro data suggests that non‐O blood type impacts the VWF glycosylation pattern and thereby alters the susceptibility of VWF to ADAMTS13‐mediated proteolysis.^38^ The impact of ABO blood type seemed to increase with age, as patients >55 years showed the highest ABO blood type‐related difference in VWF.^13^

Accordingly, with a mean age of 54.1 ± 11.4, we would have expected a profound impact of ABO blood type in our second cohort (“Vienna cohort”). In adjusted analysis, the non‐O blood type‐related increase in VWF antigen levels was about 24%, which, in absolute terms, was comparable to the differences reported in the general population.^15^ However, the median VWF level in our series of patients with portal hypertension was 313 (167)%, and thus, the relative difference was less then 10%.

High VWF levels indicate endothelial dysfunction and liver sinusoidal endothelial cells (LSEC) likely contribute to the increased VWF levels in these patients.^39^ LSEC dysfunction impacts intrahepatic resistance, and thus, aggravates portal hypertension, which may further increase bacterial translocation.^40^ In turn, bacterial translocation worsens endothelial dysfunction via toll‐like receptor 4 activation by endotoxins/lipopolysaccharides,^41^ leading to a perpetuation of these mechanisms,^42^ and, because of the intense crosstalk between parenchymal and non‐parenchymal cells in the liver, also hepatic inflammation.^43^ Finally, bacterial translocation also induces systemic inflammation and hemodynamic derangements, which may trigger acute decompensation and acute‐on‐chronic liver failure,^44^ and thus, are closely linked to mortality.^12^

In our study, VWF levels were independently and positively associated with decompensated disease, severity of portal hypertension (HVPG), indicators of hepatic dysfunction, liver injury/hepatic inflammation (AST/GGT) and systemic inflammation (CRP). This supports the above‐mentioned considerations about the interplay between hemodynamic changes (portal hypertension and hyperdynamic circulation) and hepatic/systemic inflammation leading to elevated VWF levels in patients with ACLD. Moreover, similar to the general population, age and non‐O blood type increased VWF levels, independently of the other factors. Obesity as well as the analyzed components of the MetS did not affect VWF levels in univariate analyses, and thus, were not included in multivariate models. This finding is in contrast to studies not focusing on chronic liver disease (CLD)^14^, ^45^ and may be explained by the profound effects of liver disease outweighing those of the components of the MetS. The comparison of effects of non‐O blood type and acquired CLD‐related factors in our series of patients with portal hypertension may provide an explanation for the lack of an association between ABO blood type and PVT: Although the effect of non‐O blood type on VWF levels attained statistical significance, it explained only about 1% of the variance in VWF (*r* ^2^ = .01), which is substantially less as compared to the general population (15.4%^46^). Accordingly, non‐O blood type was substantially less influential in patients with portal hypertension and predominated by acquired factors, such as HVPG (*r* ^2^ = .155) or CTP score (*r* ^2^ = .181).

Importantly, we also investigated parameters associated with factor VIII levels: VWF and factor VIII levels showed a correlation of moderate strength, which seemed to be comparable throughout groups/strata of hepatic dysfunction/portal hypertension severity. However, as a result of the limited sample size in some of the groups/strata, we cannot entirely rule out that liver disease severity modulates the correlation between VWF and factor VIII levels. Besides age, arterial hypertension, hyperlipidaemia, hepatic dysfunction, platelet count and AST were independently linked with factor VIII levels. Although factor VIII levels were not affected by BMI, overweight, or obesity, they showed an association with arterial hypertension and hyperlipidaemia (ie, components of the MetS), which is in line with observations in non‐CLD cohorts.^14^, ^45^ CRP correlated with factor VIII levels in univariate analysis, however, this association did not attain statistical significance after adjusting for the other factors. Of note, we observed no association between non‐O blood type or portal hypertension and factor VIII. This is surprising, as endothelial cells (in particular, LSEC) are considered the primary source of factor VIII^47^ and it has been shown that gut‐derived endotoxin induces the release of VWF and factor VIII by endothelial cells which form and secrete Weibel‐Palade bodies.^41^ Nevertheless, the absence of an impact of ABO blood type on factor VIII levels in our series of patients with portal hypertension may provide an additional explanation for ABO blood type not affecting PVT prevalence, since increases in factor VIII are considered as a main factor linking ABO and VTE in the general population,^48^ as well as an important contributor to the hypercoagulability paralleling liver disease progression.^49^, ^50^ Furthermore, we observed no difference in factor VIII/protein C ratio, which has previously been linked with PVT incidence in a longitudinal study.^18^

Portal vein thrombosis development in patients with ACLD has to be considered a multifactorial event not only driven by changes in coagulation, but also by reduced portal flow velocity^51^ and a series of local factors,^7^ which are unrelated to ABO blood type/coagulation. Nevertheless, there is increasing evidence linking measures of procoagulant imbalance to PVT incidence in patients with cirrhosis.^18^, ^52^

We have to acknowledge that the two main analyses included in this study were performed in different cohorts, since the “*US cohort*” did not include information on VWF/factor VIII, while in the “*Vienna cohort*,” screening for PVT was not standardised. However, the demographic characteristics of the two cohorts were comparable and both cohorts included patients with ACLD, although patients in the “*Vienna cohort*” had considerably less advanced disease. Importantly, this approach allowed to combine the strengths of the “*US cohort*” (very large sample size) and the “*Vienna cohort*” (in depth characterisation). However, the relationship between ABO blood type and PVT was evaluated in a sicker cohort than the association of ABO blood type and VWF/factor VIII levels, which is an important limitation of our study. Stratification by severity of hepatic dysfunction/portal hypertension revealed, that the impact of ABO blood type on VWF (and possibly, factor VIII) levels seemed to be limited to ACLD patients with less advanced disease (ie, patients with CTP A, subclinical portal hypertension, or MELD <10 points). As patients with these characteristics usually aren't listed for liver transplantation, they were not included in the “*US cohort*,” and thus, our findings should not be extrapolated to these patient populations. Of note, these patients with less advanced disease are per se at low risk for PVT.^7^

In conclusion, while ABO blood type contributes to the variation in VWF levels in patients with early stage ACLD, its overall impact is considerably smaller than in the general population, and may vanish in patients with advanced cirrhosis. Moreover, ABO blood type had no impact on factor VIII levels. These findings may explain the absence of an association between ABO blood type and PVT, even in a large data set of patients with advanced cirrhosis.

## CONFLICTS OF INTEREST

The authors have nothing to disclose regarding the work under consideration for publication. However, the authors disclose the following financial activities outside the submitted work: BS received travel support from AbbVie and Gilead. PQ has served as a speaker and/or consultant and/or advisory board member for Roche and Takeda. CA received honoraria for lectures and advisory boards from Bayer, Boehringer‐Ingelheim, Bristol‐Myers Squibb, Daiichi Sankyo and Pfizer. MT has served as a speaker and/or consultant and/or advisory board member for Albireo, Bristol‐Myers Squibb, Dr Falk Pharma, Gilead, Intercept, MSD, Novartis, Phenex Pharmaceuticals and Regulus, and has received research funding from Albireo, Dr Falk Pharma, Gilead, Intercept, MSD and Takeda. MT is listed as a co‐inventor on patents on the medical use of nor‐ursodeoxycholic acid. TR has served as a speaker and/or consultant and/or advisory board member for AbbVie, Bayer, Boehringer Ingelheim, Gilead, W. L. Gore & Associates and MSD and has received research funding from AbbVie, Boehringer Ingelheim, Gilead, Phenex Pharmaceuticals and Philips. MM has served as a speaker and/or consultant and/or advisory board member for AbbVie, Bristol‐Myers Squibb, Gilead, W. L. Gore & Associates and Janssen. PGN, AG, GS, GL, JT and TL have nothing to disclose.

## AUTHORS' CONTRIBUTIONS

All authors contributed either to research design (BS, TL and MM), and/or the acquisition (BS, PGN, AG, GS, GL, PQ TL and MM), analysis (BS, PGN and MM) or interpretation (all authors) of data. BS and MM drafted the manuscript, which was critically revised by all other authors.

## Supporting information

TableS1‐S7
