# Supplementary material for: The impact of ABO blood type on the prevalence of portal vein thrombosis in patients with advanced chronic liver disease
Source: Liver Int. 2020 Mar 4;40(6):1415–26. doi: 10.1111/liv.14404 (PMC7317432; doi:10.1111/liv.14404)
Supplement: Supplementary file 1 — TableS1‐S7 [file LIV-40-1415-s001.docx]

**Supplementary information**

**Supplementary table 1**

| Patient characteristics | | All patients,  n=59,292 | O,  n=26,481 | A,  n=21,902 | B,  n=7,915 | AB,  n=2,994 | *P* value |
| --- | --- | --- | --- | --- | --- | --- | --- |
| Age, years | | 52.55 ± 11.68 | 52.43 ± 11.76 | 52.59 ± 11.65 | 52.50 ± 11.59 | 53.39 ± 11.43 | 0.0003 |
| Sex | |  |  |  |  |  |  |
|  | Male | 37,546 (63.32%) | 16,707 (63.09%) | 13,999 (63.92%) | 4,949 (62.53%) | 1,891 (63.16%) | 0.1053 |
|  | Female | 21,746 (36.68%) | 9,774 (36.91%) | 7,903 (36,08%) | 2,966 (37,47%) | 1,103 (36,84%) |  |
| Overweight^1^ | | 40,347 (68.05%) | 18,086 (68.30%) | 14,976 (68.38%) | 5,284 (66.76%) | 2,001 (66.83%) | 0.0192 |
| Obese^2^ | | 20,299 (34.24%) | 9,069 (34.25%) | 7,644 (34.90%) | 2,625 (33.16%) | 961 (32.10%) | 0.0024 |
| Diabetes mellitus | | 13,564 (22.88%) | 5,932 (22.40%) | 5,003 (22.84%) | 1,917 (24.22) | 712 (23.78%) | 0.0049 |
| Etiology | |  |  |  |  |  |  |
|  | ALD | 10,288 (17.35%) | 4,522 (17.08%) | 3,934 (17.96%) | 1,322 (16.70%) | 510 (17.03%) | <0.0001 |
|  | Viral | 21,507 (36.27%) | 9,613 (36.30%) | 7,803 (35.63%) | 2,947 (37.23%) | 1,144 (38.21%) |  |
|  | NAFLD or cryptogenic | 9.834 (16.59%) | 4,302 (16.25%) | 3,777 (17.25%) | 1,256 (15.87%) | 499 (16.67%) |  |
|  |  |  |  |  |  |  |  |
|  | Other | 17,663 (29.79%) | 8,044 (30.38%) | 6,388 (29.17%) | 2,390 (30.20%) | 841 (28.09%) |  |
| MELD at listing, points | | 20.72 ± 9.64 | 21.06 ± 9.79 | 20.55 ± 9.51 | 20.48 ± 9.63 | 19.67 ± 9.20 | <0.0001 |
| MELD at transplant, points | | 23.74 ± 10.31 | 24.24 ± 10.45 | 23.70 ± 10.24 | 23.06 ± 10.14 | 21.33 ± 9.56 | <0.0001 |
| PVT at listing | | 2,521 (4.25%) | 1,087 (4.10%) | 960 (4.38%) | 339 (4.28%) | 135 (4.51%) | 0.4171 |
| PVT at transplant | | 5,024 (8.47%) | 2,223 (8.39%) | 1,873 (8.55%) | 661 (8.39%) | 267 (8.92%) | 0.7292 |

^1^BMI >25 kg x m^-2^

^2^BMI >30 kg x m^-2^

**Supplementary table 1.** Characteristics of patients in the ‘***U.S. cohort***’ stratified by ABO blood type (excluding patients with HCC or TIPS).

*Abbreviations: ALD alcoholic liver disease; BMI body mass index; HCC hepatocellular carcinoma; MELD model for end-stage liver disease; NAFLD non-alcoholic fatty liver disease; PVT portal vein thrombosis; TIPS transjugular intrahepatic portosystemic shunt*

**Supplementary table 2**

| Patient characteristics | | All patients,  n=84,947 | O,  n=37,860 | A,  n=31,509 | B,  n=11,397 | AB,  n=4,181 | *P* value |
| --- | --- | --- | --- | --- | --- | --- | --- |
| Age, years | | 54.01 ± 11.11 | 53.93 ± 11.19 | 54.02 ± 11.04 | 54.07 ± 11.07 | 54.40 ± 10.95 | 0.1049 |
| Sex | |  |  |  |  |  |  |
|  | Male | 56,784 (66.85%) | 25,189 (66.53%) | 21,278 (67.53%) | 7,543 (66.18%) | 2,774 (66.35%) | 0.0114 |
|  | Female | 28,163 (33.15%) | 12,671 (33.47) | 10,231 (32.47%) | 3,854 (33.82%) | 1,407 (33.65%) |  |
| Overweight^1^ | | 58,884 (69.32%) | 26,400 (69.73%) | 21,969 (69.72%) | 7,680 (67.39%) | 2,835 (67.81%) | <0.0001 |
| Obese^2^ | | 29,334 (34.53% | 13,216 (34.91%) | 11,021 (34.98%) | 3,759 (32.98%) | 1,338 (32.00%) | <0.0001 |
| Diabetes mellitus | | 21,239 (25.00%) | 9,340 (24.67%) | 7,843 (24.89%) | 2,972 (26.08%) | 1,084 (25.93%) | 0.0099 |
| Etiology | |  |  |  |  |  |  |
|  | ALD | 23,894 (28.13%) | 10,659 (28.15%) | 8,705 (27.63%) | 3,277 (28.75%) | 1,253 (29.97%) | <0.0001 |
|  | Viral | 11,603 (13.66%) | 5,075 (13.40%) | 4,454 (14.14%) | 1,497 (13.14%) | 577 (13.80%) |  |
|  | NAFLD or cryptogenic | 11,127 (13.10%) | 4,891 (12.92%) | 4,248 (13.48%) | 1,438 (12.62%) | 550 (13.15%) |  |
|  | HCC | 19,465 (22.91%) | 8,659 (22.87%) | 7,259 (23.04%) | 2,633 (23.10%) | 914 (21.86%) |  |
|  | Other | 18,858 (22.20%) | 8,576 (22.65%) | 6,843 (21.72%) | 2,552 (22.39%) | 887 (21.22%) |  |
| History of TIPS | | 7,412 (8.73%) | 3,283 (8.67%) | 2,798 (8.88%) | 1,013 (8.89%) | 318 (7.61%) | 0.0451 |
| MELD at listing, points | | 18.79 ± 9.50 | 19.06 ± 9.67 | 18.67 ± 9.37 | 18.52 ± 9.44 | 18.00 ± 8.99 | <0.0001 |
| MELD at transplant, points | | 21.73 ± 10.51 | 22.17 ± 10.70 | 21.72 ± 10.44 | 21.06 ± 10.30 | 19.63 ± 9.49 | <0.0001 |
| PVT at listing | | 3,802 (4.48%) | 1,654 (4.37%) | 1,431 (4.54%) | 518 (4.55%) | 199 (4.76%) | 0.5223 |
| PVT at transplant | | 8,014 (9.43%) | 3,620 (9.56%) | 2,951 (9.37%) | 1,057 (9.27%) | 386 (9.23%) | 0.6979 |

^1^BMI >25 kg x m^-2^

^2^BMI >30 kg x m^-2^

**Supplementary table 2.** Patient characteristics of the ‘***U.S. cohort***’ stratified by ABO blood type (including patients with HCC and/or TIPS).

*Abbreviations: ALD alcoholic liver disease; BMI body mass index; HCC hepatocellular carcinoma; MELD model for end-stage liver disease; NAFLD non-alcoholic fatty liver disease; PVT portal vein thrombosis; TIPS transjugular intrahepatic portosystemic shunt*

**Supplementary table 3**

| Patient characteristics | | All patients,  n=84,947 | O,  n=37,860 | Non-O,  n=47,087 | | *P* value |
| --- | --- | --- | --- | --- | --- | --- |
| Age, years | | 54 ±11.1 | 53.9 ±11.2 | 54.1 ±11 | | 0.3104 |
| Sex | | | | | | |
|  | Male | 56,784 (66.85%) | 25,189 (66.53%) | 31,595 (67.10%) | | 0.0809 |
|  | Female | 28,163 (33.15%) | 12,671 (33.47%) | 15,492 (32.90%) | |  |
| Overweight^1^ | | 58,884 (69.32%) | 26,400 (69.73%) | 32,484 (68.99%) | | 0.0195 |
| Obese^2^ | | 29,334 (34.53%) | 13,216 (34.91%) | 16,118 (34.23%) | | 0.0391 |
| Diabetes mellitus | | 21,239 (25.00%) | 9,340 (24.67%) | 11,899 (25.27%) | | 0.0446 |
| Etiology | | | | | | |
|  | Viral | 23,894 (28.13%) | 10,659 (28.15%) | 13,235 (28.11%) | <0.0001 | |
|  | ALD | 11,603 (13.66%) | 5,075 (13.40%) | 6,528 (13.86%) |  |  |
|  | NAFLD or cryptogenic | 11,127 (13.10%) | 4,891 (12.92%) | 6,236 (13.24%) |  |  |
|  | HCC | 19,465 (22.91%) | 8,659 (22.87%) | 10,806 (22.95%) |  |  |
|  | Other | 18,858 (22.20%) | 8,576 (22.65%) | 10,282 (21.84%) |  |  |
| History of TIPS | | 7,412 (8.73%) | 3,283 (8.67%) | 4,129 (8.77%) | 0.6169 | |
| Time on the waiting list | | 222 ±390 | 232 ±401 | 214 ±380 | <0.0001 | |
| MELD at listing, points | | 18.79 ±9.50 | 19.06 ±9.67 | 18.58 ±9.36 | <0.0001 | |
| MELD at transplant, points | | 21.73 ±10.51 | 22.17 ±10.70 | 21.38 ±10.34 | <0.0001 | |
| PVT at listing | | 3,802 (4.48%) | 1,654 (4.37%) | 2,148 (4.56%) | 0.1762 | |
| PVT at transplant | | 8,014 (9.43%) | 3,620 (9.56%) | 4,394 (9.33%) | 0.2546 | |

^1^BMI >25 kg x m^-2^

^2^BMI >30 kg x m^-2^

**Supplementary table 3.** Comparison of patient characteristics between patients with O and non-O blood types in the ‘***U.S. cohort***’ (including patients with HCC and/or TIPS).

*Abbreviations: ALD alcoholic liver disease; BMI body mass index; HCC hepatocellular carcinoma; MELD model for end-stage liver disease; NAFLD non-alcoholic fatty liver disease; PVT portal vein thrombosis; TIPS transjugular intrahepatic portosystemic shunt*

**Supplementary table 4**

| Group | CTP A | | | CTP B | | | CTP C | | |
| --- | --- | --- | --- | --- | --- | --- | --- | --- | --- |
| Blood type | O, n=20 | non-O, n=44 | *P* value | O, n=95 | non-O, n=164 | *P* value | O, n=32 | non-O, n=56 | *P* value |
| VWF, % | 233 (138) | 260 (136) | 0.210 | 284 (182) | 298 (152) | 0.188 | 380 (114) | 420 (67) | 0.084 |
| Stratum | MELD <10 points | | | MELD 10-15 points | | | MELD >15 points | | |
| Blood type | O, n=42 | non-O, n=97 | *P* value | O, n=67 | non-O, n=107 | *P* value | O, n=38 | non-O, n=60 | *P* value |
| VWF, % | 226 (109) | 266 (129) | 0.005 | 323 (152) | 317 (153) | 0.784 | 353 (180) | 413 (84) | 0.043 |
| Stratum | HVPG 6-9 mmHg | | | HVPG 10-15 mmHg | | | HVPG ≥16 mmHg | | |
| Blood type | O, n=13 | non-O, n=28 | *P* value | O, n=35 | non-O, n=67 | *P* value | O, n=99 | non-O, n=169 | *P* value |
| VWF, % | 199 (71) | 252 (91) | 0.019 | 213 (128) | 253 (135) | 0.052 | 332 (134) | 361 (144) | 0.108 |

**Supplementary table 4.** Impact ABO blood type on von Willebrand factor (VWF) levels in different Child-Turcotte-Pugh score (CTP), model for end-stage liver disease (MELD), and hepatic venous pressure gradient (HVPG) groups/strata of the ‘***Vienna cohort***’.

**Supplementary table 5**

| Group | CTP A | | | CTP B | | | CTP C | | |
| --- | --- | --- | --- | --- | --- | --- | --- | --- | --- |
| Blood type | O,  n=20 | non-O,  n=43 | *P* value | O,  n=64 | non-O,  n=102 | *P* value | O,  n=24 | non-O,  n=47 | *P* value |
| Factor VIII, % | 176 (68) | 205 (77) | 0.097 | 188 (115) | 198 (86) | 0.8 | 218 (46) | 201 (101) | 0.56 |
| Stratum | MELD <10 points | | | MELD 10-15 points | | | MELD >15 points | | |
| Blood type | O,  n=27 | non-O,  n=70 | *P* value | O,  n=55 | non-O,  n=78 | *P* value | O,  n=26 | non-O,  n=44 | *P* value |
| Factor VIII, % | 183 (57) | 202 (84) | 0.178 | 189 (87) | 193 (95) | 0.807 | 231 (135) | 203 (81) | 0.605 |
| Stratum | HVPG 6-9 mmHg | | | HVPG 10-15 mmHg | | | HVPG ≥16 mmHg | | |
| Blood type | O,  n=8 | non-O,  n=19 | *P* value | O,  n=24 | non-O,  n=45 | *P* value | O,  n=76 | non-O,  n=128 | *P* value |
| Factor VIII, % | 164 (90) | 237 (111) | 0.163 | 181 (75) | 193 (66) | 0.323 | 202 (78) | 196 (89) | 0.375 |

**Supplementary table 5.** Impact ABO blood type on factor VIII levels in different Child-Turcotte-Pugh score (CTP), model for end-stage liver disease (MELD), and hepatic venous pressure gradient (HVPG) groups/strata of the ‘***Vienna cohort***’.

**Supplementary table 6**

| Group | CTP A,  n=63 | | CTP B,  n=166 | | CTP C,  n=71 | |
| --- | --- | --- | --- | --- | --- | --- |
|  | ρ | *P* value | ρ | *P* value | ρ | *P* value |
| VWF – factor VIII | 0.363 | 0.003 | 0.514 | <0.001 | 0.396 | 0.001 |
| Stratum | MELD <10,  n=97 | | MELD 10-15,  n=133 | | MELD >15,  n=70 | |
|  | ρ | *P* value | ρ | *P* value | ρ | *P* value |
| VWF – factor VIII | 0.392 | <0.001 | 0.522 | <0.001 | 0.456 | <0.001 |
| Stratum | HVPG 6-9 mmHg,  n=27 | | HVPG 10-15 mmHg,  n=69 | | HVPG ≥16 mmHg,  n=204 | |
|  | ρ | *P* value | ρ | *P* value | ρ | *P* value |
| VWF – factor VIII | 0.653 | <0.001 | 0.412 | <0.001 | 0.526 | <0.001 |

**Supplementary table 6.** Correlation between factor VIII and von Willebrand factor (VWF) in different Child-Turcotte-Pugh score (CTP), model for end-stage liver disease (MELD), and hepatic venous pressure gradient (HVPG) strata/groups in the ‘***Vienna cohort***’.

**Supplementary table 7**

| Patient characteristics | | ‘***U.S. cohort***’  n=59,292 | ‘***Vienna cohort***’,  n=411 | *P* value |
| --- | --- | --- | --- | --- |
| Age, years | | 52.5 ± 11.7 | 54.1 ± 11.4 | 0.167 |
| Sex | |  |  |  |
|  | Male | 37,546 (63.32%) | 286 (69.59%) | 0.009 |
|  | Female | 21,746 (36.68%) | 125 (30.41%) |  |
| Overweight^1^ | | 40,347 (68.05%) | 237 (57.66%) | <0.0001 |
| Obese^2^ | | 20,299 (34.24%) | 88 (21.41%) | <0.0001 |
| Diabetes mellitus | | 13,564 (22.88%) | 92 (22.38%) | 0.813 |
| Etiology | |  |  |  |
|  | Viral | 21,507 (36.27%) | 156 (37.96%) | 0.479 |
|  | ALD | 10,288 (17.35%) | 144 (35.04%) | <0.0001 |
|  | NAFLD or cryptogenic | 9,834 (16.59%) | 69 (16.79%) | 0.912 |
|  | Other | 17,663 (29.79%) | 42 (10.22%) | <0.0001 |
| MELD at listing/HVPG measurement | | 20.7 ± 9.6 | 11.7 ± 4.0 | <0.0001 |

^1^BMI >25 kg x m^-2^

^2^BMI >30 kg x m^-2^

**Supplementary table 7.** Comparison of patient characteristics between the ‘***U.S. cohort***’ (excluding patients with HCC and/or TIPS) and the ‘***Vienna cohort***’.

*Abbreviations: ALD alcoholic liver disease; BMI body mass index HVPG hepatic venous pressure gradient; MELD model for end-stage liver disease; NAFLD non-alcoholic fatty liver disease*
